# Supplementary material for: Experiences of mothers and health workers with MomCare and SafeCare bundles in Kenya and Tanzania: A qualitative evaluation
Source: PLoS One. 2023 Nov 16;18(11):e0294536. doi: 10.1371/journal.pone.0294536 (PMC10653531; doi:10.1371/journal.pone.0294536)
Supplement: S1 File — (DOCX) [file pone.0294536.s001.docx]

**S1 File: Codebook**

| **Theme: Maternal experiences** | |
| --- | --- |
| **Sub-theme: Antenatal care** | **Quotes** |
| Easy access to maternal health services | *‘What I can say about MomCare is that it is a nice program. It helps women who cannot even afford ANC, SBA, and PNC to have free services. There are some services MomCare also assists women with, sometimes they assist the women who require to undergo cesarean section (IDI, Kenya, Mother).*  *‘Upon reaching the 4th month, I used to come every week because the baby was not in a good position (IDI, Tanzania, Mother).* |
| Early and full ANC visits | *‘I came when my pregnancy was four months. I came because I was suspecting that I am pregnant. I was sick, and I was vomiting a lot. I could not eat anything or even take water. I was just vomiting, and the vomit resembled that of malaria. That is why I went to the hospital then I was told that I did not have malaria, I was pregnant. I was then told to start attending the clinic and I didn’t waste time, the following week I started the clinic (IDI, Kenya, Mother).* |
| No financial constraints during the pregnancy journey | *‘Free treatment. You would come to antenatal care for free, delivery was free and also after delivery, they were giving us small gifts for free (IDI, Kenya, Mother).*  *“We used to pay for an ultrasound even if you go to a bigger hospital but now if you wanted an ultrasound you go to the clinic unit, they sign for you and get checked for free so we benefited in many things so I would like to request they should improve for us even more” (FGD, Tanzania, Mother).* |
| Respectful care | *Sometimes, I fail to come. But, here, they are very gentle to us, and also, they have good services (FGD, Kenya, Mother).*  *‘What has motivated me in this facility is that they are just perfect. I have not been to a hospital like this, the place is clean, good services. They just serve you well, with respect. (IDI, Tanzania, Mother).* |
| **Sub-theme: Skilled birth attendance (SBA) or labor and delivery** | |
| Improved laboratory testing | *‘Before going to the maternity ward, you have to get tested for COVID-19, HIV and… I was offered many tests and from there, they checked the position of the baby first because mine was done through elective CS (cesarean section) then, I was taken for surgery (FGD, Kenya, Mother).*  *‘MomCare brought all the tests now we can test mothers with all the required tests such as the HB (hemoglobin), blood level, syphilis, HIV, urinalysis, and blood group. When the mother comes for the first time, she has to get tested until when she is in labor. You monitor her during the clinic visits until she delivers. So, we have to do the tests that I told you earlier like HB (hemoglobin), urinalysis, VDRL (Venereal Disease Research Laboratory testing for syphilis), blood group, and ultrasound, although ultrasound is the last test.’ (IDI, Tanzania, Health worker)* |
| Good quality care during childbirth | *‘I started here at Mukumu because I delivered all my children here. I have been coming to clinics here and also, even though I come from far, I prefer here because it has these services such as constant checking of the babies breathing, heart beating, the mother's condition, and even CS (cesarean section) if needed (IDI, Kenya, Mother).*  *When I arrived, the doctor put on gloves, he told me to lie on the bed, he amained my belly, after that, he was able to check if the cervix had dilated that is when he told me I must wait a bit, I had to do some exercise here. Together with one nurse we went around and came back. During the day she ensured I ate well, that night we slept here (at the facility) with the nurses, the second day they woke me up at five o’clock we went for some exercise, and at six o’clock they examined me, they told me that my cervix had dilated, they encouraged me to take some tea and on that morning at six o’clock I delivered (IDI, Tanzania, Mother).* |
| **Sub-theme: Postnatal care** | |
| Sufficient health education and good care | *‘Immediately after delivery, they clean you and then they give you an injection to stop the bleeding and then you dress up and go to the resting bed. They observe you and if your status is okay, you are discharged the next day. You are told to go home. They give you a date to come for clinic and if the baby did not get the BCG vaccine and then there is the child’s medical card. You follow up on that.’ (IDI, Tanzania, Mother)* |
| Dispensing of all prescribed medications | *“The services we accessed during MomCare were much improved because we were well-considered in that, whenever the facility ran out of drugs, they were replenished on time. When I was not on the program (MomCare), I had to wait for the drugs to come from home by which time, I would have suffered because that took the time (FGD, Tanzania).* |
| **Theme: Health worker experiences** | |
| New opportunity to provide quality care | *‘Basically, it (MomCare) has allowed us, the health workers, to provide quality health services to these women and babies through lab tests, ultrasound, and also medication like we have to give some medication in the process of labor and when they go home (IDI, Kenya, Health worker)* |
| Adherence to the standard of care | *‘Because of Momcare, after a mother has delivered, she is now in PNC. We shall give her folate and vitamin K. You will also give her some eye ointment for the baby to prevent eye infections. You will observe her for 24 hours to see if there is going to be any challenge or not. After you confirm that the mother is in good health, you can release her to go home. You will give her appointments to come after 7 days and 21 days.’(IDI, Tanzania, Mother).* |
| Positive and fulfilling practice | ‘*That mother had four pregnancies and all of them were dying before delivery. The one we delivered now is the fourth. She had pressure, she had fibroids, and so on. So, the staff here began moving with her from day one of conception. So, when she reached six months, she was more in danger because children used to die between five, six, and seven months. So, the doctors decided to operate. They delivered the baby at six months. We put it in our New Born Unit. Both survived and we are happy we helped. (IDI, Kenya, Health worker).* |
| **Theme: Experiences at the health system level** | |
| Emergency and continual care | *‘There are big changes. MomCare has helped to reduce maternal deaths since it has supported those with low income, who could not afford some of the costs related to child delivery. Women come here without any cash, but the MomCare package caters to their needs. If there is any minor need for further medication, the facility usually top-ups. The same is done even in the case of surgery.” (IDI, Tanzania, Health worker).* |
| Improved infrastructure, medical supplies, and logistics | *‘It (MomCare) has helped a lot by improving infrastructure that is offering delivery services, it has helped to build family planning facilities, and it has helped in buying maternity equipment and drugs. Those (health workers) who were offering services to the women also received allowances.’ (IDI, Tanzania, Health worker).*  *‘In the health facility, generally, our maternity has improved, and we have also improved our postnatal wards because these are the major areas, even the antenatal. We had to move from the other side and come to this side because of the number of clients and at least we have a space for all of them to be accommodated (IDI, Tanzania, Health Worker).* |
| Improved staffing | *‘So, as a facility (health facility), I think we had less personnel by then, but the ones we have now are helping us run because we have hired five nurses, one clinical officer, and then of course, a lab tech (IDI, Kenya, Health worker).* |
| Increased documentation | *‘So, for negatives, I would only say that it (MomCare) had paperwork. It (MomCare) had a lot of paperwork that I did not like as a person and even the women because you see here, many people, don't even know how to write. They don't even know how to sign. So immediately they're treated, or they come for the ANC, there is a sheet that they were signing and giving their phone numbers and other details and the women disliked that even though we were assisting, you cannot assist a person to put her signature (IDI, Kenya, Health worker).* |
