# Supplementary material for: Experiences of mothers and health workers with MomCare and SafeCare bundles in Kenya and Tanzania: A qualitative evaluation
Source: PLoS One. 2023 Nov 16;18(11):e0294536. doi: 10.1371/journal.pone.0294536 (PMC10653531; doi:10.1371/journal.pone.0294536)
Supplement: Attachment — Submitted filename: PONE-D-23-14716_reviewer.pdf [file pone.0294536.s002.pdf]

PLOS ONE

Experiences of mothers and health workers with a Care Bundle in Kenya and Tanzania: a qualitative evaluation

--Manuscript Draft--

Manuscript Number:	PONE-D-23-14716
Article Type:	Research Article
Full Title:	Experiences of mothers and health workers with a Care Bundle in Kenya and Tanzania: a qualitative evaluation
Short Title:	Experiences with Care bundle in Kenya and Tanzania: a qualitative evaluation
Corresponding Author:	Jonathan Izudi, MPH, Ph.D.<br>Mbarara University of Science and Technology<br>Mbarara, UGANDA
Keywords:	Antenatal care, Care bundle, Maternal health, MomCare, Postnatal care, Skilled birth attendance
Abstract:	Between 2019 and 2022, the digital dividend project (DDP), a technology-based intervention that combined a care bundle (MomCare) and a quality improvement bundle (SafeCare) to empower mothers to access care during pregnancy, labor and delivery, and postnatally, was implemented in Kenya and Tanzania. The ultimate goal is improved maternal and newborn health outcomes. We describe the experiences of mothers in accessing and using health services under MomCare, and the experiences of the health workers in providing the services. We conducted a qualitative evaluation across health facilities in Kenya and Tanzania enrolled in MomCare. We held Interviews with mothers and health workers at the antenatal care (ANC), skilled birth attendance (SBA), and postnatal care (PNC) service delivery points. We performed content analysis and reported our findings using themes along with quotes from the participants. We studied 127 mothers (76 in Kenya, 51 in Tanzania) and 119 health workers. Our findings revealed that mothers had easy access to health services, had early and full ANC attendance, respectful care, had no financial constraints, received good quality care and all needed medications, and sufficient health education. Health worker experiences included a new opportunity to provide quality maternal and newborn care, adherence to the standard of care, and positive and fulfilling practice. On the health systems front, improvements were reported regarding emergency response and continual care, infrastructure including medical supplies and logistics, staffing, and increased documentation. Overall, MomCare strengthened the healthcare system to deliver quality maternal and child health services. We recommend the replication of MomCare in settings with similar maternal and child health challenges in sub-Saharan Africa and beyond.
Order of Authors:	Jonathan Izudi, MPH, Ph.D.
	Henry Odero Owoko, MA
	Moussa Bagayoko, MPH, Ph.D.
	Damazo Kadengye
Additional Information:	
Question	Response
Financial Disclosure<br><br>Enter a financial disclosure statement that describes the sources of funding for the work included in this submission. Review the submission guidelines for detailed requirements. View published research articles from *PLOS ONE* for specific	The evaluation was supported by Children's Investment Fund (CIFF), Kenya. The funder had no role in study design, data collection and analysis, decision to publish, or preparation of the manuscript.

Powered by Editorial Manager® and ProduXion Manager® from Aries Systems Corporation

examples.<br><br>This statement is required for submission and **will appear in the published article** if the submission is accepted. Please make sure it is accurate.<br><br>**Unfunded studies**<br>Enter: *The author(s) received no specific funding for this work.*<br><br>**Funded studies**<br>Enter a statement with the following details:<br>• Initials of the authors who received each award<br>• Grant numbers awarded to each author<br>• The full name of each funder<br>• URL of each funder website<br>• Did the sponsors or funders play any role in the study design, data collection and analysis, decision to publish, or preparation of the manuscript?<br>• **NO** - Include this sentence at the end of your statement: *The funders had no role in study design, data collection and analysis, decision to publish, or preparation of the manuscript.*<br>• **YES** - Specify the role(s) played.<br><br>* typeset	
**Competing Interests**<br><br>Use the instructions below to enter a competing interest statement for this submission. On behalf of all authors, disclose any competing interests that could be perceived to bias this work—acknowledging all financial support and any other relevant financial or non-financial competing interests.<br><br>This statement is required for submission and **will appear in the published article** if the submission is accepted. Please make sure it is accurate and that any funding sources listed in your Funding Information later in the submission form are also declared in your Financial Disclosure statement.	The authors have declared that no competing interests exist.

View published research articles from *PLOS ONE* for specific examples.<br><br>**NO authors have competing interests**<br><br>Enter: *The authors have declared that no competing interests exist.*<br><br>**Authors with competing interests**<br><br>Enter competing interest details beginning with this statement:<br><br>*I have read the journal's policy and the authors of this manuscript have the following competing interests: [insert competing interests here]*<br><br>* typeset	
**Ethics Statement**<br><br>Enter an ethics statement for this submission. This statement is required if the study involved:<br><br>• Human participants<br>• Human specimens or tissue<br>• Vertebrate animals or cephalopods<br>• Vertebrate embryos or tissues<br>• Field research<br><br>Write "N/A" if the submission does not require an ethics statement.<br><br>General guidance is provided below. Consult the submission guidelines for detailed instructions. **Make sure that all information entered here is included in the Methods section of the manuscript.**	We received ethical review and approval from the African Population and Health Research Center (APHRC) Internal Ethics Committee. The African Medical Research Foundation Ethical and Scientific Review Committee or AMREF-ESRC provided external review and ethical approval in Kenya (reference number: P911-2020). In Tanzania, the National Institute for Medical Research or NIMR (reference number: NIMR/HQ/R.8a/Vol.IX/3689) provided ethical clearance. All ethical approvals preceded the evaluation and all participants provided written informed consent.

Powered by Editorial Manager® and ProduXion Manager® from Aries Systems Corporation

**Format for specific study types**<br><br>**Human Subject Research (involving human participants and/or tissue)**<br>• Give the name of the institutional review board or ethics committee that approved the study<br>• Include the approval number and/or a statement indicating approval of this research<br>• Indicate the form of consent obtained (written/oral) or the reason that consent was not obtained (e.g. the data were analyzed anonymously)<br><br>**Animal Research (involving vertebrate animals, embryos or tissues)**<br>• Provide the name of the Institutional Animal Care and Use Committee (IACUC) or other relevant ethics board that reviewed the study protocol, and indicate whether they approved this research or granted a formal waiver of ethical approval<br>• Include an approval number if one was obtained<br>• If the study involved *non-human primates*, add *additional details* about animal welfare and steps taken to ameliorate suffering<br>• If anesthesia, euthanasia, or any kind of animal sacrifice is part of the study, include briefly which substances and/or methods were applied<br><br>**Field Research**<br><br>Include the following details if this study involves the collection of plant, animal, or other materials from a natural setting:<br>• Field permit number<br>• Name of the institution or relevant body that granted permission	
**Data Availability**<br><br>Authors are required to make all data underlying the findings described fully available, without restriction, and from the time of publication. PLOS allows rare exceptions to address legal and ethical concerns. See the PLOS Data Policy and FAQ for detailed information.	Yes - all data are fully available without restriction

A Data Availability Statement describing where the data can be found is required at submission. Your answers to this question constitute the Data Availability Statement and **will be published in the article**, if accepted.<br><br>**Important:** Stating 'data available on request from the author' is not sufficient. If your data are only available upon request, select 'No' for the first question and explain your exceptional situation in the text box.<br><br>Do the authors confirm that all data underlying the findings described in their manuscript are fully available without restriction?	
**Describe where the data may be found in full sentences. If you are copying our sample text, replace any instances of XXX with the appropriate details.**<br><br>• If the data are **held or will be held in a public repository**, include URLs, accession numbers or DOIs. If this information will only be available after acceptance, indicate this by ticking the box below. For example: *All XXX files are available from the XXX database (accession number(s) XXX, XXX.).*<br>• If the data are all contained **within the manuscript and/or Supporting Information files**, enter the following: *All relevant data are within the manuscript and its Supporting Information files.*<br>• If neither of these applies but you are able to provide **details of access elsewhere**, with or without limitations, please do so. For example:<br><br>*Data cannot be shared publicly because of [XXX]. Data are available from the XXX Institutional Data Access / Ethics Committee (contact via XXX) for researchers who meet the criteria for access to confidential data.*<br><br>*The data underlying the results presented in the study are available from (include the name of the third party*	All relevant data are within the manuscript and its Supporting Information files.

and contact information or URL).<br>• This text is appropriate if the data are owned by a third party and authors do not have permission to share the data.<br><br>* typeset	
Additional data availability information:	

Manuscript

Click here to access/download;Manuscript;Digitial Divident Manuscript [006].docx

Full title

Experiences of mothers and health workers with a Care Bundle in Kenya and Tanzania: a qualitative evaluation

Short title

Experiences with Care bundle in Kenya and Tanzania: a qualitative evaluation

Authors

[1*]Jonathan Izudi, [1]Henry Odero Owoko, [1]Moussa Bagayoko, [1]Damazo Kadengye

Author affiliations

1. African Population and Health Research Center (APHRC), Nairobi, Kenya.

*Corresponding author

Email: jonahzd@gmail.com (JI)

Email: jizudi@aphrc.org (JI)

Abstract

Between 2019 and 2022, the digital dividend project (DDP), a technology-based intervention that combined a care bundle (MomCare) and a quality improvement bundle (SafeCare) to empower mothers to access care during pregnancy, labor and delivery, and postnatally, was implemented in Kenya and Tanzania. The ultimate goal is improved maternal and newborn health outcomes. We describe the experiences of mothers in accessing and using health services under MomCare, and the experiences of the health workers in providing the services. We conducted a qualitative evaluation across health facilities in Kenya and Tanzania enrolled in MomCare. We held Interviews with mothers and health workers at the antenatal care (ANC), skilled birth attendance (SBA), and postnatal care (PNC) service delivery points. We performed content analysis and reported our findings using themes along with quotes from the participants. We studied 127 mothers (76 in Kenya, 51 in Tanzania) and 119 health workers. Our findings revealed that mothers had easy access to health services, had early and full ANC attendance, respectful care, had no financial constraints, received good quality care and all needed medications, and sufficient health education. Health worker experiences included a new opportunity to provide quality maternal and newborn care, adherence to the standard of care, and positive and fulfilling practice. On the health systems front, improvements were reported regarding emergency response and continual care, infrastructure including medical supplies and logistics, staffing, and increased documentation. Overall, MomCare strengthened the healthcare system to deliver quality maternal and child health services. We recommend the replication of MomCare in settings with similar maternal and child health challenges in sub-Saharan Africa and beyond.

Introduction

Limited access to and non-use of maternal and child health services contribute to significant maternal and newborn morbidity and mortality. Globally, an estimated 810 women die daily from preventable causes related to pregnancy and childbirth, with 99% of the deaths being in sub-Saharan Africa (SSA) where access to quality maternal and child health services are limited[1]. The direct causes of maternal mortality, namely excessive blood loss, infection, high blood pressure, unsafe abortion, and obstructed labor [1], and the indirect causes like anemia, malaria, tuberculosis, human immunodeficiency virus (HIV), and heart diseases among others[2] are all preventable and treatable if access to quality antenatal care (ANC), skilled birth attendance (SBA), and postnatal care (PNC) are guaranteed. The risk of under-five mortality in SSA is 15 times higher than in developed regions despite a declining global trend[3], with the leading causes as preterm delivery, pneumonia, birth asphyxia, diarrhea and malaria, and malnutrition among others. These causes are equally preventable and treatable with access to quality child health services.

Both Kenya and Tanzania have poor maternal and newborn outcomes. The 2022 Demographic and Health Survey data placed the maternal mortality ratio (MMR) in Kenya at 342 deaths per 100,000 live births, and in Tanzania at 524 deaths per 100,00 live births. To achieve the Sustainable Development Goal or SDG target of reducing MMR to less than 70 deaths per 100,000 live births and neonatal mortality to below 12 deaths per 1,000 live births, context-specific interventions are urgently needed. One such intervention is to use digital technologies in health. For example, mobile phone technologies (sometimes called telehealth, telemedicine, e-health, or mhealth) have emerged as promising tools to improve access and use of maternal and child health services, with the ultimate goal of improving maternal and child health outcomes[4] Digital technologies remove barriers to accessing maternal health services by ensuring economic and geographic convenience[5]. Digital technologies promote health in general but also health education, health management, and health research. Digital technologies improve access to health care[6], with the added benefits of increased knowledge due to the availability of information about pregnancy and newborn health[7]. Digital technologies have been reported to increase ANC visits and improve the timing and quality of ANC services[8-10]. Telemedicine and phone-based referral networks have been recommended as solutions to address declines in the availability and use of PNC services during and after the recent COVID-19 lockdown[11].

By supplementing in-person visits with mobile applications, mental health outcomes during pregnancy became comparable or even better, and the replacement of in-person visits with reduced prenatal care visits using telehealth for low-risk pregnancies led to similar clinical outcomes and high patient satisfaction with care[12]. Recent systematic review and meta-analysis showed that remote breastfeeding support using digital technologies significantly reduces the risk of exclusive breastfeeding cessation at 3 months by 25%[13].

In 2019, the MMR in Kenya was 362 deaths per 100,000 live births[14], and that in Tanzania was 578 deaths per 100,000 live births[15]. The 5-year neonatal mortality rate (NNMR) was 22 per 1,000 live births and the infant mortality rate (IMR) was 39 per 1,000 live births[16]. In Tanzania, the NNMR was 25 deaths per 1,000 births and the IMR was 43 deaths per 1,000 births at the time[17]. These rates are among the highest in SSA. A digital dividend project, a technology-based intervention that combined a care bundle (MomCare) and quality improvement bundle (SafeCare), was therefore started in Kenya and Tanzania to improve access to quality maternal health services during pregnancy, labor and delivery, and postnatal periods, with the overall goal of improving maternal and newborn health outcomes. The project intended to achieve the outcomes through 1) contracting health workers to provide quality care for women; 2) assessing and improving the quality of care provided through SafeCare standards and tools; 3) monitoring health facilities for quality of care to ensure optimal pregnancy journey at the best cost; 4) providing women with the means to save or access subsidies including insurance and top-ups to pay for health services (the health wallet), and 5) rewarding health workers with bonuses in recognition for quality services that meet all pre-agreed criteria for healthcare delivery. To date, little is known about the experiences of mothers and health workers with MomCare. Here, we described the experiences of mothers in accessing and utilizing MomCare during antenatal care, labor and delivery, and postnatal care. We also described the experiences of health workers in providing health services to mothers, both in Kenya and Tanzania between 2019 and 2022.

Methods and materials

Description of MomCare

Between 2019 and September 2022, a three-year digital dividend project was implemented in Tanzania and Kenya by PharmAccess Foundation International, with funding from the Children's Investment Fund Foundation (CIFF). The project combined a care bundle (MomCare) and a quality improvement bundle (SafeCare) to empower women to access the care they trust throughout their pregnancy journey and the postnatal period. Women were enrolled in a subsidized health insurance program and check-in and all the clinic costs were paid using a mobile platform. The service included "nudges" to remind women regarding check-ups and rewards to improve commitment. At the end of the care journey, health workers are compensated financially for providing high-quality care. Also, health workers were financially rewarded for positive health outcomes at the end of the care journey thus directly incentivizing quality and ensuring patient-centered care. The care journey for a mother begins at no more than 26 weeks of gestation except for teenagers and women living with human immunodeficiency virus (HIV), who could be enrolled at any point during the journey. The enrolment ended at 20 weeks post-delivery, marking the 60th week of the care journey. Each step of the care journey was digitally tracked, and data were collected and analyzed to track and improve healthcare delivery. SafeCare package is an internationally recognized standard-based quality improvement and recognition approach that has been operational since 2010[18]. To improve the efficiency and cost-effectiveness of the SafeCare process, a digital assessment tool was developed. With this tool, SafeCare provides each health facility with a SafeCare quality score on a scale of 1-5 whenever a mother uses a service followed by a discussion of an automated quality improvement plan on the same day. In addition, the tool has automatic cross-checks allowing a reduced need and time for manual assessment and reviews by supervisors.

Study design and setting

This qualitative evaluation was conducted in the two East African countries of Kenya and Tanzania between November and December 2022. In Kenya, 16 health facilities in Kisumu and Kakamega counties were included and in Tanzania, there were 51 health facilities from the Manyara and Kilimanjaro regions. In both countries, the regions selected had high maternal and neonatal morbidity and mortality based on the respective Demographic and Health Survey (DHS) data.

Besides, the regions had suboptimal use of ANC, SBA, and PNC services. For example, in Tanzania, the nomadic nature of the communities limited the use of existing services due to financial and physical constraints. In Kenya, the regions had the worst maternal and child health indicators, with MMR at 495 deaths per 100,000 live births and IMR at 40 deaths per 1000 live births in Kakamega and 50 deaths per 1000 live births in Kisumu. In both settings, data were collected among participants at the ANC, SBA, and PNC service delivery points.

Study population

We studied women aged 19-49 years who had benefited from MomCare and health workers who provided MomCare at the respective health facilities in Kenya and Tanzania. The women were purposively sampled based on the maximum variance sampling method, an approach that allowed equal representation of all women based on residence (rural versus urban), parity, insurance status, receipt of incentives, and level of education. The same sampling approach was applied to health workers (physicians, nurses, and midwives), health facility level, and years of experience (<5 versus ≥5 years). At the managerial and leadership levels, we interviewed the health facility In-Charges, and for each of the categories of health workers, a minimum of five were included.

Data collection

Data were collected through in-depth interviews (IDI) with mothers and health workers, and a focused group discussion (FGD) with mothers. Interviews were conducted in a noiseless place within the health facility premise, Monday to Friday, 9.00 am to 5.00 pm. The data collection focused on the experiences of mothers as beneficiaries of MomCare and the health workers' experiences in providing maternal and child health services under MomCare. IDIs were conducted in the local language "*Kiswahili*" with the women and in the English language with health workers. FGDs were conducted by two people, a moderator, and a note taker, and each group comprised 8-12 women with comparable age and parity.

Quality control measures

Research assistants had ≥3 years of qualitative research experience and were drawn from the study area to ensure a better understanding of the local context and to ease the data collection.

All research assistants are trained in the health sciences discipline, namely nursing, clinical medicine, community health, and health nutrition. They received a 5-day training on the evaluation design, data collection process, and responsible conduct of research. Research assistants were organized into teams, each comprised of five people with one as the Team Leader. The Team Leader tracked the progress of the teams daily and provided technical assistance to the teams but with the support of the evaluation team. All data collection tools were pre-tested at a distant health facility not included in the evaluation. The feedback from pre-testing the tools was used to improve the implementation of the evaluation.

Statistical issues

Sample size estimation

No sample size was calculated but the number of people interviewed depended on a priori sample size deemed sufficient to achieve saturation, a point at which no new information emerges despite additional data collection. Our a priori sample size was 210 mothers (105 Kenya, 105 Tanzania) and approximately 150 health workers (75 Kenya, 75 Tanzania). We achieved saturation with 127 mothers (76 in Kenya, 51 in Tanzania) and 119 health workers.

Data analysis

Data were collected through voice recordings and thereafter transcribed verbatim. The transcripts were verified by replaying the voice recordings. Any disparities between the transcript and the voice recordings were corrected. Field notes were scrutinized and compared with the audio recordings and the transcripts were cross-referenced with the field notes. Areas of departure were highlighted and discussed between the analysts. Transcriptions were done by 10 research assistants with experience in qualitative research. Dedoose software version 9.0.54 was used for the data analysis. The analysis was conducted by two independent female analysts to prevent subjective bias, and each analyst had ≥10 years of experience in qualitative research. The analysts coded the transcripts independently and developed the initial codes that were later harmonized through discussions and consensus to form the final codebook. The initial codes were then applied to the rest of the transcripts. The analysis adopted a thematic content approach and followed three steps, namely data immersion, coding, and coding sort. In data immersion, the two analysts (SJ and MN) familiarized themselves with the transcripts by reading and re-reading the transcripts several times to identify common and important texts and patterns. They allowed impressions to shape the data interpretations in different and unpredictable directions.

SJ and MN then flagged the relevant parts of the transcripts with suitable words or codes and in the final stages, both analysts categorized the codes into themes and sub-themes in the agreed codebook. Three senior reviewers (MB, DK, and JI), with experience in qualitative and mixed-methods research, verified all emergent codes, themes, and sub-themes including the final codebook to minimize subject bias. The main themes and sub-themes were presented along with the participant's quotes.

Ethical consideration

We received ethical review and approval from the African Population and Health Research Center (APHRC) Internal Ethics Committee. The African Medical Research Foundation Ethical and Scientific Review Committee or AMREF-ESRC provided external review and ethical approval in Kenya (reference number: P911-2020). In Tanzania, the National Institute for Medical Research or NIMR (reference number: NIMR/HQ/R.8a/Vol.IX/3689) provided ethical clearance. All ethical approvals preceded the evaluation and all participants provided written informed consent. Number tags and pseudo names were used during data collection. Participation in the study was entirely voluntary and withdrawal from participation was permissible at any time.

Results

Characteristics of the participants

We summarize the participant's characteristics in Table 1. Overall, 127 mothers were included in the study, mainly from Kenya (n=76) but not Tanzania (n=51). The majority of the participants were from a rural setting, with parity ≥2, secondary or more levels of education, and at the PNC clinic. Health workers were mainly in the nursing and midwifery professions combined (n= 105).

Table 1: Participant Characteristics and Distributions

Variables	Levels	Kenya (n=76)	Tanzania (n=51)	Overall (n=127)
**Mothers**				
Residence	Urban	21	18	39
	Rural	44	29	73
	Peri-urban	11	4	15
Parity	1	26	10	36
	≥2	50	41	91
Level of education	None	2	1	3
	Primary	23	35	58
	Secondary and over	51	15	66
Insurance status	Yes	50	15	65
	No	26	36	62
Point of service delivery	ANC	22	25	47
	SBA	21	4	25
	PNC	33	22	55
**Health workers**	**Levels**			
Type of health worker (n=119)	Nurses and midwives	62	43	105
	Physician	14	0	14

Main findings

We present the findings under three main themes ─ experiences at maternal, health worker, and health systems levels. Table 2 presents the emergent sub and main themes. We present the experiences of mothers based on the services received during ANC, SBA, and PNC. Health worker experiences have been presented under three sub-themes and experiences at the health system level were summarized under four sub-themes.

Table 2: Summary of main themes and sub-themes.

Main themes	Sub-themes
**Maternal experiences**	**Antenatal care**
	1. Easy access to maternal health services
	2. Early and full ANC visits
	3. No financial constraints during the pregnancy journey
	4. Respectful care
	**Skilled birth attendance (SBA) or labor and delivery**
	1. Improved laboratory testing
	2. Good quality care during childbirth
	**Postnatal care**
	1. Sufficient health education and good care
	2. Dispensing of all prescribed medications
**Health worker experiences**	1. New opportunity to provide quality care
	2. Adherence to the standard of care
	3. Positive and fulfilling practice
**Experiences at the health system level**	1. Emergency and continual care
	2. Improved infrastructure, medical supplies, and logistics
	3. Improved staffing
	4. Increased documentation

Maternal experiences during antenatal care (ANC)

Easy access to maternal health services

Participants reported MomCare led to improved access to ANC services. Many of the participants described the ANC service as of good quality. The participants were happy with the care bundle because it has led successful pregnancy journey. Success was mentioned as the absence of complications during pregnancy.

> *'What I can say about MomCare is that it is a nice program. It helps women who cannot even afford ANC, SBA, and PNC to have free services. There are some services MomCare also assists women with, sometimes they assist the women who require to undergo cesarean section (IDI, Kenya, Mother).*

‘MomCare is a good thing because there was a time I came when sick and got admitted like today and discharge the following day and I used it. Unlike others (insurance), you will be told that if you use it once, then you cannot use the card again for the second time that is why I like MomCare(IDI, Kenya, Mother).

‘Upon reaching the 4th month, I used to come every week because the baby was not in a good position (IDI, Tanzania, Mothe[illegible]

Early and full ANC visits

The participants reported having received their first ANC visit between the first and the fifth month of pregnancy, with the majority indicating to have started their first ANC visits in the fourth and third months of pregnancy. A few participants reported a late first ANC visit, which was either in the sixth or seventh month of pregnancy.

‘I came when my pregnancy was four months. I came because I was suspecting that I am pregnant. I was sick, and I was vomiting a lot. I could not eat anything or even take wate[illegible] I was just vomiting, and the vomit resembled that of malaria. That is why I went to the hospital then I was told that I did not have malaria, I was pregnant. I was then told to start attending the clinic and I didn’t waste time, the following week I started the clinic (IDI, Kenya, Mother).

Participants indicated attending all scheduled ANC visits, with many reporting four to five ANC visits and a few reporting two to three ANC visits due to late first ANC attendance. Participants with high-risk pregnancies attended as many as 7-9 ANC as they needed close monitoring.

‘It (ANC visits) used to be every month depending on the date that you were scheduled. Let’s say today is the 30th, you might be scheduled to come back next month on the 23rd. It depends. Every month you go (ANC visit) depending on the return date that you have been given (IDI, Kenya, Mother).

‘I came for the test, and they told me I am pregnant, then I went back and waited for four months before I started going to the clinic (meaning ANC clinic) many times, I think 5 to 6 times, yes. (IDI, Tanzania, Mother).

No financial constraints during the pregnancy journey

Participants mentioned that MomCare eased access to ANC, SBA, and PNC as the services became free insurance due to schemes like the Linda Mama and the National Health Insurance Fund. In addition, they reported that the insurance scheme provided comprehensive cover for their pregnancy journey like more than one free ultrasound scan and several laboratory tests and other procedures.

> *'I did not make any payments in the hospital. You know a hospital like Port Florence is private. When you go there, you will pay a lot of money but, when you have that card or NHIF (National Health Insurance Fund) you do not pay for anything (IDI, Kenya, Mother).*

> *'Free treatment. You would come to antenatal care for free, delivery was free and also after delivery, they were giving us small gifts for free (IDI, Kenya, Mother).*

> *"We used to pay for an ultrasound even if you go to a bigger hospital but now if you wanted an ultrasound you go to the clinic unit, they sign for you and get checked for free so we benefited in many things so I would like to request they should improve for us even more" (FGD, Tanzania, Mother).*

Respectful care

It emerged that the health workers treated the mothers with respect and dignity during ANC visits, a factor that motivated them to even continue with all planned ANC schedules. The health workers also had a positive attitude towards pregnant women during their ANC visits.

> *'Me, what motivates me is the way I am being treated when I come for services. If you ask people about their experiences some will tell you they gave birth in a certain hospital, they were abused by nurses or if they are late, they were quarreled at so I also came with that fear of being quarreled at when I am late because I come from far.*

> *Sometimes, I fail to come. But, here, they are very gentle to us, and also, they have good services (FGD, Kenya, Mother).*

> *'What has motivated me in this facility is that they are just perfect. I have not been to a hospital like this, the place is clean, good services. They just serve you well, with respect. (IDI, Tanzania, Mother).*

Maternal experiences during SBA or labor and delivery

Improved laboratory testing

Participants said the health workers performed several tests on them to check if they were at risk for complications during their pregnancy or delivery. Notable tests performed included those for HIV, COVID-19, syphilis, hepatitis, and high blood sugar level, among others.

> *'Before going to the maternity ward, you have to get tested for COVID-19, HIV and… I was offered many tests and from there, they checked the position of the baby first because mine was done through elective CS (cesarean section) then, I was taken for surgery (FGD, Kenya, Mother).*

> *'MomCare brought all the tests now we can test mothers with all the required tests such as the HB (hemoglobin), blood level, syphilis, HIV, urinalysis, and blood group. When the mother comes for the first time, she has to get tested until when she is in labor. You monitor her during the clinic visits until she delivers. So, we have to do the tests that I told you earlier like HB (hemoglobin), urinalysis, VDRL (Venereal Disease Research Laboratory testing for syphilis), blood group, and ultrasound, although ultrasound is the last test.' (IDI, Tanzania, Health worker).*

Good quality care during childbirth

It was stated that the quality of health services at the Momcare health facilities was good so many of the participants chose to receive both ANC and SBA services at the same health facility. MomCare health facilities were recommended to other participants because of good quality care and friendly health workers during SBA, and easy access and affordable health services.

> *'I started here at Mukumu because I delivered all my children here. I have been coming to clinics here and also, even though I come from far, I prefer here because it has these services such as constant checking of the babies breathing, heart beating, the mother's condition, and even CS (cesarean section) if needed (IDI, Kenya, Mother).*

> '*When I arrived, the doctor put on gloves, he told me to lie on the bed, he amained my belly, after that, he was able to check if the cervix had dilated that is when he told me I must wait a bit, I had to do some exercise here. Together with one nurse we went around and came back. During the day she ensured I ate well, that night we slept here (at the facility) with the nurses, the second day they woke me up at five o'clock we went for some exercise, and at six o'clock they examined me, they told me that my cervix had dilated,*

they encouraged me to take some tea and on that morning at six o'clock I delivered (IDI, Tanzania, Mother).

Maternal experiences during PNC

Sufficient health education and good care

Complications during the PNC period are unpredictable both for the mother and the newborn so information on when to seek help is important. The participants indicated that the health workers provided them with sufficient health information during PNC visits to ensure their safety and that of the newborns.

> *'After delivery, they continue to educate you on how to bathe the baby, then tell you to breastfeed the baby for six months (FGD, Kenya).*

> *'Immediately after delivery, they clean you and then they give you an injection to stop the bleeding and then you dress up and go to the resting bed. They observe you and if your status is okay, you are discharged the next day. You are told to go home. They give you a date to come for clinic and if the baby did not get the BCG vaccine and then there is the child's medical card. You follow up on that.' (IDI, Tanzania, Mother)*

Dispensing of all prescribed medications

A sufficient supply of medications or drugs is an important component of a strong health system. Through MomCare, all required medications during PNC became available. Participants indicated they received all the required medications during PNC visits.

> *"All medications will be given to this mother as also immunization services we offer. In case the child is aged between 0-14 weeks we treat this child and also process referrals if we have an emergency to refer the mother for further management, we just refer using our ambulance because it also helps the mother, it (MomCare) covers ambulance services (IDI, Kenya, Mother).*

> *"The services we accessed during MomCare were much improved because we were well-considered in that, whenever the facility ran out of drugs, they were replenished on time. When I was not on the program (MomCare), I had to wait for the drugs to come from home by which time, I would have suffered because that took the time (FGD, Tanzania).*

Healthcare provider experiences

New opportunity to provide quality care

Health workers indicated that MomCare presented them with a new opportunity to provide quality health services to mothers and their newborns. They felt motivated to provide all needed services as the care bundle had incentives like training, regular support supervision, and follow-up by the implementing partners.

> *‘Basically, it (MomCare) has allowed us, the health workers, to provide quality health services to these women and babies through lab tests, ultrasound, and also medication like we have to give some medication in the process of labor and when they go home (IDI, Kenya, Health worker)*

> *'It (meaning allowances) encourages a worker (meaning a health worker) who can do extra work, or extra hours to work. We have a few staff. So MomCare has truly helped to enlighten us.' (IDI, Tanzania, Health worker).*

Adherence to the standard of care

Health workers indicated that MomCare was less restrictive, allowing them to adhere to the standards of care at all times. For instance, they indicated that the program permitted additional laboratory tests like urinalysis, Rhesus factor, and examinations like ultrasound scanning to be performed whenever needed.

> *‘I will manage the post-delivery process in case of a complication. I'm sure MomCare will take care of that. Then also the clients are free; the one that uses MomCare just comes in free. Even if she's sick, just comes in compared with the others (IDI, Kenya, Health Worker).*

> *‘Because of Momcare, after a mother has delivered, she is now in PNC. We shall give her folate and vitamin K. You will also give her some eye ointment for the baby to prevent eye infections. You will observe her for 24 hours to see if there is going to be any challenge or not.*

> *After you confirm that the mother is in good health, you can release her to go home. You will give her appointments to come after 7 days and 21 days.’(IDI, Tanzania, Mother).*

Positive and fulfilling practice

MomCare was regarded as a blessing to health facilities and mothers. Health workers stated the program served women from all walks of life regardless of their socioeconomic status, residence, age, and HIV status. In addition, they mentioned that by providing high-quality care to the mothers, they experienced a positive and fulfilling practice since all the mothers had positive outcomes at the end of their pregnancy journey.

> *'That mother had four pregnancies and all of them were dying before delivery. The one we delivered now is the fourth. She had pressure, she had fibroids, and so on. So, the staff here began moving with her from day one of conception. So, when she reached six months, she was more in danger because children used to die between five, six, and seven months. So, the doctors decided to operate. They delivered the baby at six months. We put it in our New Born Unit. Both survived and we are happy we helped. (IDI, Kenya, Health worker).*

Experiences at the health system level

Emergency and continual care

MomCare was considered an inclusive health service delivery by health workers. Health workers mentioned that MomCare led to a fast response to emergencies whenever needed thus reducing the risk of maternal deaths.

> *'………. they (mothers) do benefit in terms of emergencies. If you have a mother who is under MomCare and maybe she is in the village and goes into labor, and then she calls, we usually provide an ambulance. Do you see that as an advantage that others (mothers not covered by MomCare) would not get? (IDI, Kenya, Health Worker).*

> *'There are big changes. MomCare has helped to reduce maternal deaths since it has supported those with low income, who could not afford some of the costs related to child delivery. Women come here without any cash, but the MomCare package caters to their needs. If there is any minor need for further medication, the facility usually top-ups. The same is done even in the case of surgery." (IDI, Tanzania, Health worker).*

‘*When it comes to delivery, even at one time, it came a time when the program realized that there are women that time there was during COVID time (COVID-19), movements were restricted they provided ambulance services and women were ferried as long as they would call, the hospital would provide an ambulance and as long as is it is confirmed it is a MomCare mother they pay for it then, in maternity as they deliver, all those services and the expenses that she would incur the MomCare would cater for their payments (IDI, Kenya, Health worker)*.

Improved infrastructure, medical supplies, and logistics

Health workers reported that MomCare guidelines helped to improve the health facility infrastructure as well as the availability of medical equipment and supplies. Such improvements have been reported to have not only reduced inter-health facility referrals but increased positive outcomes and created a conducive workplace.

‘*Facility (Health facility) has signage all over that will give you directions. You will know this is MCH (Maternal and Child Health Clinic), that is accounts, and that is administration, all courtesy of MomCare. The facility walls were also painted and the iron sheets were painted afresh. That was in 2019 it was courtesy of MomCare. The computers, most are courtesy of MomCare. Sometimes staff takes tea once in a while, courtesy of MomCare. A lot has happened. We have a new theatre, courtesy of MomCare. It's because of the increase in the number of clients (women) that the new theatre had to be built (IDI, Kenya, Health worker).*

‘It (MomCare) has helped a lot by improving infrastructure that is offering delivery services, it has helped to build family planning facilities, and it has helped in buying maternity equipment and drugs. Those (health workers) who were offering services to the women also received allowances.’ (IDI, Tanzania, Health worker).

‘In the health facility, generally, our maternity has improved, and we have also improved our postnatal wards because these are the major areas, even the antenatal. We had to move from the other side and come to this side because of the number of clients and at least we have a space for all of them to be accommodated (IDI, Tanzania, Health Worker).

Improved staffing

Understaffing was a common problem at health facilities before MomCare was introduced. Health workers stated that MomCare nurses and midwives were hired to keep pace with the growing number of deliveries.

‘So, as a facility (health facility), I think we had less personnel by then, but the ones we have now are helping us run because we have hired five nurses, one clinical officer, and then of course, a lab tech (IDI, Kenya, Health worker).

Increased documentation

Health workers noted that the project involved a lot of paperwork, especially during registration and since the majority of the women were illiterate, the process even became much harder.

‘So, for negatives, I would only say that it (MomCare) had paperwork. It (MomCare) had a lot of paperwork that I did not like as a person and even the women because you see here, many people, don't even know how to write. They don't even know how to sign. So immediately they're treated, or they come for the ANC, there is a sheet that they were signing and giving their phone numbers and other details and the women disliked that even though we were assisting, you cannot assist a person to put her signature (IDI, Kenya, Health worker).

Discussion

We report maternal experiences in accessing and using antenatal, skilled birth, and postnatal care, and the experiences of health workers in providing the services during MomCare in Kenya and Tanzania. Our findings revealed positive experiences with MomCare among mothers and health workers. Maternal experiences revealed easy access to health services, early and full ANC attendance, respectful care, absence of financial constraints, good quality care, receipt of all needed medications, and sufficient health education. Findings from health workers revealed a new opportunity to provide quality maternal and newborn care, adherence to the standard of care, and positive and fulfilling practice. On the health systems front, improvements were found in emergency response and continual care, infrastructure, medical supplies and logistics, staffing, and increased documentation at all three service delivery points.

Our findings of positive experiences among mothers and health workers are not surprising as several studies report digital technologies to improve the utilization of antenatal, skilled birth, and postnatal care. Digital health technologies are increasingly being used in several sub-Saharan African countries to improve maternal and child health services. Notable health services being improved using digital health technologies include ANC, SBA, and PNC among others[19].

One study conducted in Southern Tanzania showed that a digital health intervention improved neonatal healthcare outcomes, namely temperature control by keeping the neonates dry and warm, cord-cutting practices among health workers, and breastfeeding practices among mothers, including better preparation for obstetric care among expectant mothers through birth preparedness and complication readiness plans[19]. Our findings are consistent with the previous studies. Our findings are also in agreement with a previous study conducted in Tanzania that showed the use of digital health solutions for high-risk pregnancies improves the identification of women at risk for obstetric complications and the subsequent referral to higher-level health facilities[20].

We found MomCare led to positive experiences among health workers regarding service provision, which is not surprising. One previous study conducted about health workers' knowledge and attitudes towards the use of digital technologies in the provision of maternal health services at Tumbi regional referral hospital in Tanzania found increased use of digital health technologies[21]. The study further showed that health workers understood the importance of digital health technologies in improving maternal health services besides reporting a positive attitude towards digital health technologies[21]. In another Tanzanian study, a mobile job aid was successfully used to support the counseling of women about contraception[22], which is consistent with our findings about the better quality of care during the postnatal care period due to MoMcare. Our findings agree with the increasing use of digital health technologies to eliminate barriers to accessing health services in sub-Saharan Africa[5].

We found a strong health system due to MomCare concerning improvements in maternal and neonatal health services delivery, increased staffing (human resources for health) for the delivery of maternal and neonatal health services, strengthening of the supply of health commodities (sufficient drugs, supplies, and equipment) combined with infrastructural development, and increased financing for maternal and neonatal health services delivery, all consistent with the World Health Organization’s (WHO's) framework for health systems strengthening[23].

The WHO argues that a strong health system produces the desired quantity and quality of health services, has an adequate number of skilled health workers of an optimal mix, receives sufficient funding to support health service delivery, has a strong medical logistics and supply chain, and has a strong health management information system. With the implementation of MomCare all six WHO's health systems strengthening blocks remarkably improved.

Study strengths and limitations

The study strengths include a large sample size, data collection from all categories of participants, and all three service delivery points (ANC, SBA, and PNC). Limitations include a lack of baseline qualitative data for comparison of the present findings and the use of a qualitative evaluation that cannot demonstrate causation.

Conclusion and recommendations

The implementation MomCare strengthened the health system for maternal and child health services delivery. Maternal experiences regarding access and utilization of ANC, SBA, and PNC were largely positive. Health worker experiences revealed satisfaction with health services delivery and a positive and fulfilling practice. We recommend the replication of MomCare in settings with similar maternal and child health challenges in SSA and beyond.

Acknowledgments

We thank the Children Investment Fund Foundation (CIFF) and PharmAccess International for supporting this evaluation. We are overwhelmingly indebted to the study participants for their time in providing invaluable information. We are grateful to all research assistants for their support.

References

1. Onambele L, Ortega-Leon W, Guillen-Aguinaga S, Forjaz MJ, Yoseph A, Guillen-Aguinaga L, et al. Maternal Mortality in Africa: Regional Trends (2000-2017). International journal of environmental research and public health. 2022;19(20). Epub 2022/10/28. doi: 10.3390/ijerph192013146. PubMed PMID: 36293727; PubMed Central PMCID: PMCPMC9602585.

2. Kurjak A, Stanojević M, Dudenhausen J. Why maternal mortality in the world remains tragedy in low-income countries and shame for high-income ones: will sustainable development goals (SDG) help? Journal of perinatal medicine. 2023;51(2):170-81. Epub 2022/06/01. doi: 10.1515/jpm-2022-0061. PubMed PMID: 35636412.

3. Chamie J. The Dying Children Divide. Population Levels, Trends, and Differentials: More Important Population Matters: Springer; 2023. p. 301-4.

4. Feroz A, Perveen S, Aftab W. Role of mHealth applications for improving antenatal and postnatal care in low and middle income countries: a systematic review. BMC health services research. 2017;17(1):704. Epub 2017/11/09. doi: 10.1186/s12913-017-2664-7. PubMed PMID: 29115992; PubMed Central PMCID: PMCPMC5678803.

5. Bilal W, Mohanan P, Rahmat ZS, Ahmed Gangat S, Islam Z, Essar MY, et al. Improving access to maternal care in Africa through telemedicine and digital health. The International journal of health planning and management. 2022;37(4):2494-500. Epub 2022/05/17. doi: 10.1002/hpm.3498. PubMed PMID: 35570361.

6. Heřman H, Faridová A, Tefr O, Farid S, Ayayee N, Trojanová K, et al. Telemedicine in prenatal care. Central European journal of public health. 2022;30(2):131-5. Epub 2022/07/26. doi: 10.21101/cejph.a7458. PubMed PMID: 35876602.

7. Kusyanti T, Wirakusumah FF, Rinawan FR, Muhith A, Purbasari A, Mawardi F, et al. Technology-Based (Mhealth) and Standard/Traditional Maternal Care for Pregnant Woman: A Systematic Literature Review. Healthcare (Basel, Switzerland). 2022;10(7). Epub 2022/07/28. doi: 10.3390/healthcare10071287. PubMed PMID: 35885813; PubMed Central PMCID: PMCPMC9322765.

8. Jo Y, Labrique AB, Lefevre AE, Mehl G, Pfaff T, Walker N, et al. Using the lives saved tool (LiST) to model mHealth impact on neonatal survival in resource-limited settings. PloS one. 2014;9(7):e102224. Epub 2014/07/12. doi: 10.1371/journal.pone.0102224. PubMed PMID: 25014008; PubMed Central PMCID: PMCPMC4094557.

9. Lund S, Nielsen BB, Hemed M, Boas IM, Said A, Said K, et al. Mobile phones improve antenatal care attendance in Zanzibar: a cluster randomized controlled trial. BMC pregnancy and childbirth. 2014;14:29. Epub 2014/01/21. doi: 10.1186/1471-2393-14-29. PubMed PMID: 24438517; PubMed Central PMCID: PMCPMC3898378.

10. Kaewkungwal J, Singhasivanon P, Khamsiriwatchara A, Sawang S, Meankaew P, Wechsart A. Application of smart phone in "Better Border Healthcare Program": a module for mother and child care. BMC medical informatics and decision making. 2010;10:69. Epub 2010/11/05. doi: 10.1186/1472-6947-10-69. PubMed PMID: 21047412; PubMed Central PMCID: PMCPMC2989931.

11. Dzinamarira T, Moyo E, Pierre G, Mpabuka E, Kahere M, Tungwarara N, et al. Postnatal care services availability and utilization during the COVID-19 era in sub-Saharan Africa: A rapid review. Women and birth : journal of the Australian College of Midwives. 2022. Epub 2022/10/18. doi: 10.1016/j.wombi.2022.10.002. PubMed PMID: 36253282; PubMed Central PMCID: PMCPMC9550672.

12. Cantor AG, Jungbauer RM, Totten AM, Tilden EL, Holmes R, Ahmed A, et al. Telehealth Strategies for the Delivery of Maternal Health Care : A Rapid Review. Annals of internal medicine. 2022;175(9):1285-97. Epub 2022/07/26. doi: 10.7326/m22-0737. PubMed PMID: 35878405.

13. Gavine A, Marshall J, Buchanan P, Cameron J, Leger A, Ross S, et al. Remote provision of breastfeeding support and education: Systematic review and meta-analysis. Maternal & child nutrition. 2022;18(2):e13296. Epub 2021/12/30. doi: 10.1111/mcn.13296. PubMed PMID: 34964542; PubMed Central PMCID: PMCPMC8932718.

14. Lang'at E, Mwanri L, Temmerman M. Effects of implementing free maternity service policy in Kenya: an interrupted time series analysis. BMC health services research. 2019;19(1):645. Epub 2019/09/08. doi: 10.1186/s12913-019-4462-x. PubMed PMID: 31492134; PubMed Central PMCID: PMCPMC6729061.

15. Prasad N, Mwakatundu N, Dominico S, Masako P, Mongo W, Mwanshemele Y, et al. Improving Maternal and Reproductive Health in Kigoma, Tanzania: A 13-Year Initiative. Global health, science and practice. 2022;10(2). Epub 2022/04/30. doi: 10.9745/ghsp-d-21-00484. PubMed PMID: 35487559; PubMed Central PMCID: PMCPMC9053157.

16. Magati P, Drope J, Mureithi L, Lencucha R. Socio-economic and demographic determinants of tobacco use in Kenya: findings from the Kenya Demographic and Health Survey 2014. Pan Afr Med J. 2018;30:166. Epub 2018/11/21. doi: 10.11604/pamj.2018.30.166.14771. PubMed PMID: 30455795; PubMed Central PMCID: PMCPMC6235476.

17. Woldeamanuel BT. Trends and factors associated to early initiation of breastfeeding, exclusive breastfeeding and duration of breastfeeding in Ethiopia: evidence from the Ethiopia Demographic and Health Survey 2016. International breastfeeding journal. 2020;15(1):3. Epub 2020/01/12. doi: 10.1186/s13006-019-0248-3. PubMed PMID: 31924229; PubMed Central PMCID: PMCPMC6953467.

18. Syengo M, Suchman L. Private Providers' Experiences Implementing a Package of Interventions to Improve Quality of Care in Kenya: Findings From a Qualitative Evaluation. Global health, science and practice. 2020;8(3):478-87. Epub 2020/10/04. doi: 10.9745/ghsp-d-20-00034. PubMed PMID: 33008859; PubMed Central PMCID: PMCPMC7541106.

19. Sarkar ND, Grietens KP, Dillip A. Towards a digitally-enabled, community-based responsive health system in Tanzania: a formative study for the Afya-Tek digitised health initiative. The Lancet Global Health. 2020;8:S35.

20. Gupta A, Agrawal R, Gupt A, Guleri R, Bajpayee D, Joshi N, et al. Systems E-approach for women at risk (SEWA)-A digital health solution for detection of high-risk pregnancies. Journal of family medicine and primary care. 2021;10(10):3712-9. Epub 2021/12/23. doi: 10.4103/jfmpc.jfmpc_466_21. PubMed PMID: 34934670; PubMed Central PMCID: PMCPMC8653496.

21. Thadeus WK, Mushi LD. Health care professional knowledge and attitude towards the use of digital technologies in provision of maternal health services at Tumbi regional referral hospital in Tanzania. Journal of Medical Research Innovation. 2021;5(1):e000233-e.

22. McCool J, Dobson R, Muinga N, Paton C, Pagliari C, Agawal S, et al. Factors influencing the sustainability of digital health interventions in low-resource settings: Lessons from five countries. Journal of global health. 2020;10(2):020396. Epub 2020/12/05. doi: 10.7189/jogh.10.020396. PubMed PMID: 33274059; PubMed Central PMCID: PMCPMC7696238 form (available upon request from the corresponding author), and declare no conflicts of interest.

23. World Health Organization. Everybody's business--strengthening health systems to improve health outcomes: WHO's framework for action. 2007.

Supplementary information

S1 File: Codebook.

Click here to access/download
Supporting Information
S1 File.docx
