# Supplementary material for: Experiences of mothers and health workers with MomCare and SafeCare bundles in Kenya and Tanzania: A qualitative evaluation
Source: PLoS One. 2023 Nov 16;18(11):e0294536. doi: 10.1371/journal.pone.0294536 (PMC10653531; doi:10.1371/journal.pone.0294536)
Supplement: Attachment — Submitted filename: Response to Reviewers.docx [file pone.0294536.s003.docx]

**Editorial comments:**

**Jounal Requirements:**

When submitting your revision, we need you to address these additional requirements.

1. Please ensure that your manuscript meets PLOS ONE's style requirements, including those for file naming. The PLOS ONE style templates can be found at https://journals.plos.org/plosone/s/file?id=wjVg/PLOSOne_formatting_sample_main_body.pdf and <https://journals.plos.org/plosone/s/file?id=ba62/PLOSOne_formatting_sample_title_authors_affiliations.pdf>.

**Response**. We have adhered to the PLOS ONE’s style requirements.

2. Thank you for stating in your Funding Statement: “The evaluation was supported by Children's Investment Fund (CIFF), Kenya. The funder had no role in study design, data collection and analysis, decision to publish, or preparation of the manuscript.” Please provide an amended statement that declares *all* the funding or sources of support (whether external or internal to your organization) received during this study, as detailed online in our guide for authors at http://journals.plos.org/plosone/s/submit-now. Please also include the statement “There was no additional external funding received for this study.” in your updated Funding Statement.

**Response**. We have revised the Funding Statement to incorporate the needed changes. The revised Funding Statement included in the manuscript reads “The evaluation was supported by Children's Investment Fund (CIFF), Kenya. The funder had no role in study design, data collection and analysis, decision to publish, or preparation of the manuscript. There was no additional external funding received for this study.”

1. Please include your amended Funding Statement within your cover letter. We will change the online submission form on your behalf.

**Response**. The amended Funding Statement has been included in the cover letter and it reads: “The evaluation was supported by Children's Investment Fund (CIFF), Kenya. The funder had no role in study design, data collection and analysis, decision to publish, or preparation of the manuscript. There was no additional external funding received for this study.”

3. We note that the grant information you provided in the ‘Funding Information’ and ‘Financial Disclosure’ sections do not match. When you resubmit, please ensure that you provide the correct grant numbers for the awards you received for your study in the ‘Funding Information’ section.

**Response.** We have provided a matching funding information and financial disclosure sections now. Both now reads “The evaluation was supported by Children's Investment Fund (CIFF), Kenya. The funder had no role in study design, data collection and analysis, decision to publish, or preparation of the manuscript. There was no additional external funding received for this study.”

4. Thank you for stating the following in the Acknowledgments Section of your manuscript: “We thank the Children Investment Fund Foundation (CIFF) and PharmAccess International for supporting this evaluation. We are overwhelmingly indebted to the study participants for their time in providing invaluable information. We are grateful to all research assistants for their support.” We note that you have provided additional information within the Acknowledgements Section that is not currently declared in your Funding Statement. Please note that funding information should not appear in the Acknowledgments section or other areas of your manuscript. We will only publish funding information present in the Funding Statement section of the online submission form.

Response. We have removed the funding information from the Acknowledgement Section. The remain text reads “We are overwhelmingly indebted to the study participants for their time in providing invaluable information. We are grateful to all research assistants for their support.”

2. Please remove any funding-related text from the manuscript and let us know how you would like to update your Funding Statement. Currently, your Funding Statement reads as follows: “The evaluation was supported by Children's Investment Fund (CIFF), Kenya. The funder had no role in study design, data collection and analysis, decision to publish, or preparation of the manuscript.”

Response. We have removed any funding-related text from the manuscript. Our updated funding statement should read as follows: “The evaluation was supported by Children's Investment Fund (CIFF), Kenya. The funder had no role in study design, data collection and analysis, decision to publish, or preparation of the manuscript. There was no additional external funding received for this study.”

3. Please include your amended statements within your cover letter; we will change the online submission form on your behalf.

Response. We have indicated the amended statements in the cover letter and it reads as follows: “The evaluation was supported by Children's Investment Fund (CIFF), Kenya. The funder had no role in study design, data collection and analysis, decision to publish, or preparation of the manuscript. There was no additional external funding received for this study.”

Additional Editor Comments (if provided):

1. Thank you for considering PLoS ONE for your manuscript "Experiences of mothers and health workers with a Care Bundle in Kenya and Tanzania: a qualitative evaluation". Peer review of your manuscript is now complete and, based on the reports, a major decision has been made your consideration on the shared comments on which final decision will be made. Please you can go ahead to address the reviewer’s comments and submit them within 10 working days.

**Response.** We are grateful for considering our manuscript for review and the wonderful comments from the reviewers which have improved the quality of the manuscript.

**Reviewer #1:**

Review comments on Experiences with mothers and health care givers with a care bundle in Kenya and Tanzania. The authors have made a good attempt at qualitatively evaluating a sort of mhealth-driven maternal and newborn health improvement project. The work is well written but will be significantly improved if the authors address the following concerns.

**Response.** Taking for taking time to review our manuscript. Your comments have greatly improved the quality of our manuscript. In the revised manuscript, we have strongly considered your comment #12 so we have re-arranged the findings based on the World Health Organization’s (WHO’s) health systems building blocks, namely service delivery, health work force, financing, infrastructure and commodities, heath information systems, and leadership and governance. Our findings address 5 of the 6 domains of the blocks. We hope you will find our revised manuscript more favorable.

1. Include the period of data collection in the abstract.

**Response**. We have added the data collection period and the new text reads “Between November and December 2022, we conducted a qualitative evaluation across health facilities in Kenya and Tanzania.”

2. The Introduction is well written. Lines 82-89 could be improved by quantifying the many improvements alluded to.

Response. We have re-checked the citations and noted that the importance of digital technologies was reported qualitatively so we found it hard to quantify it. We propose to maintain the description in qualitative terms. Second, we have revised the text on lines 82-89 for clarity so it reads: “The benefits of digital technologies include removing barriers to accessing maternal health services by increasing economic and geographic convenience; promoting health in general but also increasing access to health education, health management, and health research; and, increasing access to health care[6], with the added benefits of increased knowledge as information about pregnancy and newborn health become readily available. Other benefits include increasing the frequency of ANC visits, timing, and quality of services.”

3. Since much of the problem seems to centre around access, the authors are encouraged to speak more to it by adequately describing what it used to be before the digital dividend project got underway.

**Response.** Thank you for this valuable comment. Problematic access to quality maternal and child health services mostly manifested inform of poor health outcomes. For these reasons, we provided a description of the poor health outcomes before the project implementation thus justifying a need for an intervention. The text reads "In 2019, the MMR in Kenya was 362 deaths per 100,000 live births, and that in Tanzania was 578 deaths per 100,000 live births. The 5-year neonatal mortality rate (NNMR) in Kenya was 22 per 1,000 live births and the infant mortality rate (IMR) was 39 per 1,000 live births. In Tanzania, the NNMR was 25 deaths per 1,000 births and the IMR was 43 deaths per 1,000 births. These rates are among the highest in SSA".

In addition, we added a new text to emphasize the problem of access to health services and it reads " One of the reasons for the poor maternal and child health outcomes is a lack of adequate access to quality health services in certain regions within and between the countries."

4. “To date, little is known about the experiences of mothers and health workers with MomCare.”………why is this a problem? What is to be lost if this ‘problem’ remains unaddressed. The problem statement and study rationale are not well defined.

**Response**. We have revised the sentence for clarity thus providing a rationale for the evaluation of the project. The revised sentence reads as follows: “Despite the potential benefits of the care bundles, there is little known about the experiences of mothers and health workers with the bundles. Understanding these experiences is critical in order to inform the programming and implementation of the digital dividend project—recognizing the program failures and successes, determining the best approaches for addressing the failures, and taking full advantage of the successes in future programming.”

5. “Besides, the regions had suboptimal use of ANC, SBA, and PNC services”……..the authors should try to quantify this. For instance, adequate ANC coverage (at least 4 visits) was X% in ABC County compared to Y% in DEF County over the same period of time.

**Response.** Thank you for this comment. We have deleted the above sentence because it is a repeat of previous data.

6. More than 3x as many health facilities were involved in Tanzania than in Kenya. Can the authors explain why this is so?

**Response.** We agree regarding the huge difference in the number of health facilities reported in the study between Kenya and Tanzania—51 vs. 16, respectively. These figures are accurate and are based on geographical and population differences between the 2 countries, with Tanzania having a wider geographical scope and a higher population compared to Kenya. To improve access to health services for its population, it is inevitable that Tanzania had more health facilities than Kenya. Third, from a programmatic viewpoint, the project was implemented at more health facilities in Tanzania than Kenya hence the observed difference.

7. I am thinking it is better to use the participant ID codes instead of just ‘mother, Kenya’. That brings out better the individuality in the responses. Also, include the average time of the interviews and the number of FGDs conducted.

**Response.** We have included the average time for the interviews using the text “Interviews were conducted in a noiseless place within the health facility premises, Monday to Friday, 9.00 am to 5.00 pm, lasting 30-45 minutes on average”.

Regarding the number of participants in each focused group discussion (FGD), we had included it our earlier submission and the text was: “FGDs were conducted by two people, a moderator and a note-taker. Each group comprised 8-12 women with comparable age and parity”. This text has been maintained in the revised submission. Regarding using participant codes, the idea was to anonymize the data. Based on the comment, we have now added the participants unique codes.

8. Sentence 301-303 doesn’t come out well. It appears to be missing something. Look at it again.

**Response.** We agree that the sentence was not clear as some words were missed. We have revised the text for clarity and it reads “The participants mentioned that the bundles eased access to ANC, SBA, and PNC as the services became free due to health insurance schemes like the Linda Mama and the National Health Insurance Fund. In addition, they reported that the health insurance scheme provided them with comprehensive cover for their pregnancy journey like receiving more than one free ultrasound scan and undertaking several laboratory tests and other procedures.”

9. Under ‘Respectful Care’, 2 participants make mention of “good services”. Can the authors delve deeper into what constitutes “good services”?

**Response**. Thank you for this valuable comment. We reported quotes from 2 participants although several participants talked about respectful care. As stated in the quotes, the mothers were pleased with the health services they received because the health workers handled them with care, never harassed or abused them, and treated them with dignity and respect. We have revised the sentence to strengthen the point and to bring to context that several participants actually talked about respectful care.

“The majority of the women in Kenya and Tanzania indicate that the health workers treated them with respect and dignity during their ANC visits, a factor that motivated them to continue with all planned ANC schedules. For example, the health workers showed a positive attitude towards pregnant women during ANC visits, provided them with non-judgmental care, and ensured a conducive environment and open communication including respect—constituting respectful care. Overall, there was a good relationship between the health workers and mothers.”

10. The ‘Discussion’ will be better presented if the authors gave a description of what the health system and service delivery context looked like before the roll-out of MomCare. What services were available or unavailable before MomCare? That way, the readers can better situate the benefits derived from MomCare.

**Response**. We agree with this comment. We have provided a brief description of health status and health service delivery to set the pace for contrasting with the discussion points.

The brief text reads: “Before the implementation of the bundles, the majority of the mothers had shunned the health facilities as health services were of insufficient quality and access to health care was problematic due to high direct and indirect costs. In addition, the health workers were demotivated to provide care as the working environment was not conducive due to several factors: a lack of needed equipment, drug stockouts, and inadequate health staffing among others—constituting a weak health system.”

11. MomCare offered a subsidized payment on insurance premiums and it shot up utilization of services. Much as this is a qualitative work, the authors can still beef things up with quantitative hospital data regarding ANC attendance, Skilled birth delivery and PNC attendance in the three years before MomCare so that we can see the kind of transition MomCare has brought. In my country, we have had absolutely free maternal care and delivery services since 2007. We have made good progress in the proportion of pregnant women attending ANC at least once and at least 4x but we still don’t have a 100%. Let us not create the impression MomCare has led to a universal utilization of maternal and newborn services.

**Response.** We agree to not overstating the benefits of MomCare in improving service utilization as it complemented the standard of care. Therefore, we have revised certain sections of the texts. We do not have quantitative data about maternal and child health outcomes as well as the service utilization, both of which have been stated as limitations to the study. The revised limitations section read as follows: “Limitations include a lack of baseline qualitative and quantitative data for comparison of the present findings and the use of a qualitative evaluation that cannot demonstrate causation (cause-effect relationship). The lack of quantitative data about service indicators both before and after the evaluation to provide a benchmark for comparison is another limitation worth considering. Lastly, it should be noted that bundles complemented the standard of care. Therefore, the findings should be cautiously interpreted so as not to give the impression as if all outcomes are singly attributed to the bundles.”

12. Can the authors situate their findings (some if not all) in relevant theories underlying health seeking behaviour and motivation?

**Response.** We appreciate the use of theories, models, or frameworks in presenting the findings of qualitative studies because it improves the rigor. Accordingly, we have adapted the World Health Organization’s (WHO’s) health systems strengthening building blocks framework in reporting the findings. We have added a text in the methods section, under the data analysis to indicate this change. The text reads “Along with the participant’s quotes, we presented the themes and sub-themes for the maternal and health worker experiences based on the World Health Organization’s health systems strengthening building blocks as the guiding framework. The six building blocks are service delivery, health work force, financing, infrastructure and commodities, heath information systems, and leadership and governance.”

13. I am missing key challenges in the project. Were there none?

**Response.** The project indeed had challenges. However, we felt that the challenges fell outside the scope of the question addressed by the current manuscript. Therefore, we did not report them.

**Reviewer #2:**

Thank you for the opportunity to review the manuscript titled: Experiences of mothers and health workers with a Care Bundle in Kenya and Tanzania: a qualitative evaluation.

**Response.** We thank you for dedicating time to rigorously read and review our manuscript, and to provide insightful comments which have now improved the quality. We are forever grateful. Below, we addressed each specific comment and where we had challenges in addressing any, we offered and explanation.

The title:

1. You are evaluating an experience of women and health workers on two bundles; this should be well shown in the title. Mom Care and Safe Care

**Response**. We have revised the title and it reads: “Experiences of mothers and health workers with MomCare and SafeCare Bundles in Kenya and Tanzania: a qualitative evaluation”

**Abstract:**

2. The abstract is unstructured.

**Response.** The PLoS ONE requirement is to have an unstructured abstract hence the current abstract. We are happy to submit a structured abstract if required.

3. Should give the brief description of participants (? pregnant women, lactating women, type of healthcare workers)

**Response**. We have added a description of the participants. The new text reads: “We held Interviews with mothers (pregnant and postpartum women who had benefited from the care bundles) and health workers (physicians, nurses, and midwives who provided the care bundles, including health facility In-Charges) at the antenatal care (ANC), skilled birth attendance (SBA), and postnatal care (PNC) service delivery points.”

4. Themes are not clear and are too many. Should try to summaries and make them clear.

**Response**. We appreciate this comment and have revised the themes for clarity. Regarding the number of themes, we found it a little hard to reduce them given the objectives of the study. The themes and sub-themes are a summary of the important ones. Please note that reviewer #1 suggested that we used a model, or theory, or framework to situate our findings (comment #12). In response, we have used the WHO health systems building blocks as a framework to report the findings. These now reduced the themes to 5 but the sub-themes remain. We propose to maintain them.

5. Conclusion not aligned with the findings.

**Response**. Overall, the bundles led to a strengthened heath system for better delivery of maternal and child health services and our conclusion has been aligned to speak to this finding. Therefore, the text for the conclusion reads as follows: “Overall, the care bundles led to the strengthening of the healthcare system (staffing, service delivery, financing, supplies/logistics, and information management) in order to deliver quality maternal and child health services. The bundles should be replicated in settings with similar maternal and child health challenges.”

6. Overall: The abstract needs revision to be clear and focused.

**Response.** We have revised the abstract for clarity.

**Background**

7. Needs to revise and make readable with easiness, as no full stop and no space between sentence and reference.

**Response.** We have revised the background section, addressing all punctuation errors and other identifiable issues.

8. Line 75: Recheck 524 deaths per 100,00…Need to be per 100,000.

**Response.** This has been corrected to read as “524 deaths per 100,000 live births.”

9. 76 revisit the sentence, you may need to omit the word “or”

**Response**. This has been corrected. The revised text reads “To achieve the Sustainable Development Goal (SDG) target of reducing MMR to less than 70 deaths per 100,000 live births and neonatal mortality to below 12 deaths per 1,000 live births, context-specific interventions are urgently needed.”

10. Line 97-101 is the repetition of paragraph line 73 with different references. You may need to combine this to be one paragraph.

**Response.** Thank you for this comment. The initial lines reported the most recent data about maternal mortality ratio (MMR) using the 2022 data. The text is " The 2022 Demographic and Health Survey data placed the maternal mortality ratio (MMR) in Kenya at 342 deaths per 100,000 live births, and in Tanzania at 524 deaths per 100,000 live births." The other lines, reported MMR in 2019—the data that motivated a need for the project. The text is "In 2019, the MMR in Kenya was 362 deaths per 100,000 live births, and that in Tanzania was 578 deaths per 100,000 live births." Both sections are thus different.

11. Overall: Revise language and arrange the paragraphs to get better floor.

**Response**. We have revised the manuscript for clarity and performed spelling and grammatically checks.

**Methods:**

12. Move the description of Mom Care to the background.

**Response.** We have added the description of MomCare to the background as recommended.

13. Sample size: include reference to justify your method of sample size estimation.

**Response.** We have provided reference to justify the principle of saturation as used in qualitative studies. The revised text reads as follows: “No sample size was calculated but the number of people interviewed depended on a priori sample size deemed sufficient to achieve saturation, a point at which no new information emerges despite additional data collection as reported in previous qualitative studies”. Reference numbers are #19-21.

14. Line 207: Why specifically female analyst.

**Response.** We do not have a particular reason for using female analysts. It was by chance that female analysts were recruited. Overall, no specific reason.

**General comment**

15. Needs English revision.

**Response**. We have read the manuscript and made needed revisions. We have done a spelling and grammatically checks. We believe the manuscript is clear now.
